# Use of Locally Produced Novel Low-Cost 3D Printed Respiratory Muscle Strength Trainer Device (RMSTD) for Long COVID-Rehabilitation: An Innovative Case Report

**DOI:** 10.1155/2024/8877421

**Published:** 2024-09-02

**Authors:** Dipendra Kandel, Arjan J. Knulst, Joshua Riggsbee, Sarah O. Riggsbee, Suman Tamang, Himal Bikram Bhattarai, Mitesh Karn

**Affiliations:** ^1^ International Nepal Fellowship-Nepal Green Pastures Hospital and Rehabilitation Center, Pokhara, Nepal; ^2^ Department of Biomechanical Engineering Delft University of Technology, Delft, Netherlands; ^3^ School of Medicine Gandaki Medical College Teaching Hospital and Research Center, Pokhara, Nepal

## Abstract

*Introduction*. This article details the development and use of a low-cost, custom RMST device for a patient with long COVID who had received positive airway flow support during ICU treatment. By sharing our successful management of respiratory muscle weakness in a severe COVID-19 patient, we aim to contribute to the broader conversation around effective long COVID management. *Case Description*. A patient with respiratory muscle weakness used a hospital-made RMST device. The training involved ten short forced exhalations per cycle for five cycles, followed by five long forced exhalations held for five seconds. Additionally, the patient learned lower abdominal and box breathing techniques. The patient showed significant improvement, using the RMST device without supplemental oxygen within 24 hours and completely weaned off by day 5. By discharge, the patient could complete the RMST exercise regime and achieved 290 meters in the 6MWT. After six weeks of outpatient therapy, the patient used the RMST device at 80 cm H_2_O and increased their 6MWT distance to 390 meters. *Device Description*. The RMST device was designed for in-house production with a 3D-printed PETG cap, base, and plunger, and a standard pen spring. Its design mimicked a standard PEEP valve with a different geometry. The spring closed the valve until a pressure threshold was reached, allowing airflow. The adjustable pressure threshold ranged from −40 to +40 cm H_2_O, calibrated in 10 cm H_2_O increments by measuring displaced water column height. *Discussion and Limitation*. COVID-19 can cause long-term respiratory issues needing proper management and rehabilitation. Inspiratory muscle training benefits those recovering from severe COVID-19 who were weaned from mechanical ventilation. However, the device's design and production method are unsuitable for large-scale and commercial production. Rehabilitation centers should prepare for postintensive care syndrome in post-COVID-19 individuals, with interprofessional teams addressing various recovery aspects. Early medical attention and therapy can improve patients' quality of life.

## 1. Introduction

Those symptoms of acute COVID-19 persisting even after 4 weeks of infection are termed as long COVID [1]. Post-COVID-19 syndrome is multifactorial and has multiorgan consequences, varying from physical and cognitive impairment to functional limitation, leading to a decrease in quality of life [2]. A wide range of pulmonary symptoms, including exertional dyspnea, restrictive pulmonary physiology, and decreased diffusion capacity, as well as fibrotic lung lesions on high resolution computed tomography (HRCT), have been recorded in up to 35% of survivors and are directly related to the severity of the acute illness [3]. The management of long-term consequences using a multidisciplinary approach is an important prerequisite [2]. While a growing body of evidence suggests that a well-designed pulmonary rehabilitation program may benefit patients with long-term dyspnea, fatigue, and exercise limitation, the ideal treatment approach for other persistent imaging, functional, and clinical symptoms is less apparent [4].

COVID-19 infection can lead to significant respiratory muscle weakness, a novel and concerning aspect of the disease's long-term sequelae. This weakness may contribute to both acute and persistent dyspnea in affected patients, underscoring the importance of addressing respiratory muscle dysfunction as part of their recovery. Therefore, respiratory muscle training becomes a critical intervention, offering potential benefits for improving respiratory function and alleviating symptoms in patients who continue to experience breathing difficulties postinfection [1, 5]. Incentive spirometry (IS), pressure threshold devices, and resistance devices are a few respiratory muscle strength trainers that can be used to increase the strength and endurance of respiratory muscles such as the intercostal and diaphragm [6]. The devices available on the market for respiratory muscle training were expensive, making them inaccessible to many patients in need in our setting. Recognizing this challenge, we saw the necessity to develop our own low-cost device that could be easily affordable and accessible to all patients. We ensured that effective respiratory muscle training is within reach for everyone, particularly those who might otherwise be unable to afford the commercially available options. Herein, we describe a case of an adult male with long COVID that was managed by integrated approach with speech therapy and use of a novel low-cost locally produced 3D printed respiratory training device.

### 1.1. Device description

**Figure 1:**
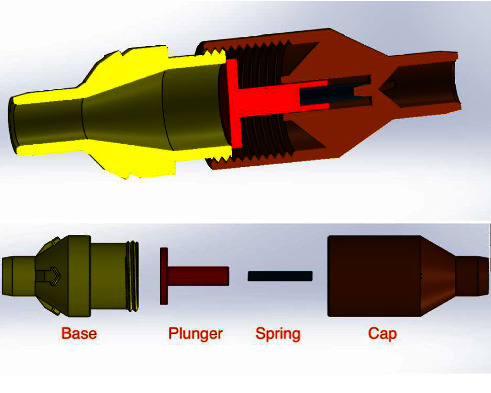
Cross-section of 2nd generation RMT prototype. Valve body (yellow), valve plunger (red), spring (dark blue), and cap (brown).

**Figure 2:**
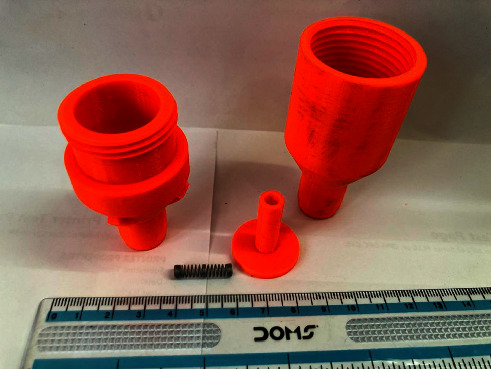
2nd generation RMT prototype showing valve body, valve plunger, spring, and cap.

**Figure 3:**
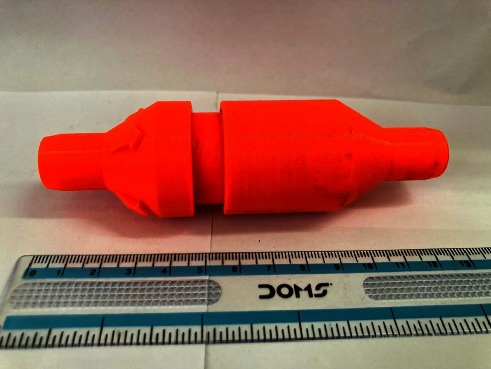
2nd generation RMT prototype. Arrows on the body indicate the direction of flow for expiratory use.

A respiratory training device was designed for in-house production consisting of a 3D printed PETG (Polyethylene Terephthalate Glycol) cap, base, and plunger, and a standard spring of a pen (Figures 1, 2, 3). The design was similar to that of a standard PEEP (Positive End-Expiratory Pressure) valve but in a different geometry. A spring closes the valve until the pressure threshold is reached and then it opens to allow airflow. The devices were printed on a Prusa mk3 printer and sliced on PrusaSlicer using default PETG printing settings and 0.10 mm layer height. The adjustable pressure threshold ranged from −40 to +40 cm H_2_O. Calibration was performed on the 0 to +40 cm H_2_O range in 10 cm H_2_O increments measuring the displaced water column height.

## 2. History and Intervention

**Figure 4:**
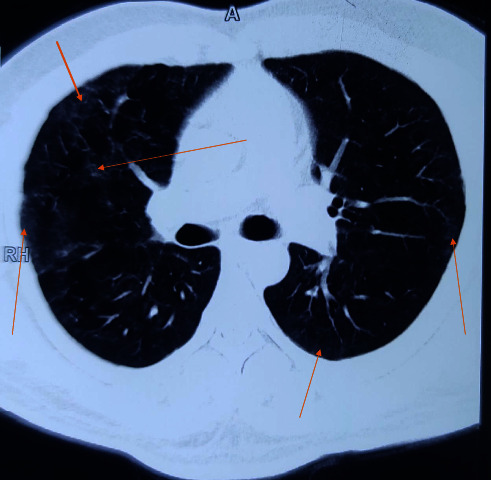
Initial HRCT of the chest (arrows indicate fibrosis of the bilateral lungs).

A 36-year-old male presented with difficulty in breathing, overwhelming fatigue, reduced range of movement, breathlessness, and dysfunctional breathing pattern. He was a known case of severe COVID-19 infection and needed intensive care unit (ICU) admission during his infective episode five months back. He was managed conservatively and kept on 15 liters per minute (LPM) of oxygen through nonbreather for 27 days as he refused mechanical ventilation. He was assessed by the Speech Language Pathologist (SLP) on the day of admission in our center (Green Pastures Hospital and Rehabilitation Centre). The individual was on two LPM oxygen via nasal cannula, with resting SpO2 of 93% and a respiratory rate of 22–25 breaths per minute. He had a clavicular breathing pattern with little diaphragm movement. When he completed any activities, such as serial swallows, sustained vocalization, or conversational tasks, his SpO2 dropped below 90% and he required 30 seconds to 2 minutes of recovery breathing. His sustained vocalization was 3 seconds. The patient was diagnosed with steroid stress hyperglycemia which was managed clinically. His Fasting Blood Sugar (FBS) was 230 mg/dl, Post-Prandial Blood Sugar (PPBS) was 290 mg/dl, but glycated hemoglobin (HBA1C) was 5.6%. He was given metformin in divided doses, and on discharge date, his FBS was 162 mg/dl and PPBS was 260 mg/dl. We continued his metformin and counseled about diabetic diet (healthy food habit and lifestyle modification). At the end of 6-week therapy along with metformin, his sugar levels returned to the baseline. The medical team performed HRCT of the chest which revealed extensive fibrosis of the bilateral lungs (Figure 4).

After his initial assessment, the SLP provided the patient with a hospital made RMST. The patient's initial maximum expiratory pressure was less than 10 cm H_2_O. Using the standard practice of exercise completion at 75% of maximum effort, the RMST device was set at 10 cm H_2_O displaced. He was taught to complete ten short forced exhalations per cycle with a total of five cycles and then five long forced exhalations holding for five seconds. He was taught to complete these cycles hourly and as needed. The SLP taught the patient lower abdominal (diaphragmatic breathing) and box breathing techniques to slow the respiratory rate. By the end of the SLP evaluation, the patient's respiratory rate had slowed to 15–17 breaths per minute and SpO2 had increased to 96% while using the RMSTD.

Over the 6 next days of hospital admission, the individual presented with rapid and remarkable improvement. During admission, he was unable to perform a 6-minute walk test (6MWT) with the Borg scale [7, 8] of 19-20. Within 24 hours of admission, he was able to use the RMST device without supplemental oxygen and maintain SpO2 of 90%. Supplemental oxygen was completely weaned on day 5 of admission. By discharge on day 7, he was independently using thoracic and diaphragmatic breathing techniques, while at rest, he was able to complete RMST device exercise regime at 40 cm H_2_O displaced with twenty repetitions per cycle and had sustained breathing of 15 seconds still below normal, but it was much improved. During discharge, the patient was able to achieve 290 m of 6MWT within the Borg scale [7, 8] of 13–15 while maintaining all the vitals within normal limits except for SpO2 which was lowest recorded 83% with Borg scale [7, 8] of 15 at the end of 6MWT. He quickly recovered with 2 minutes of rest with SpO2 maintained at greater than 93%.

When the individual returned as an outpatient, he completed two additional speech therapy sessions while exercising in the wellness gym. He was taught to self-monitor for diaphragmatic breathing and pursed lip exhalation during times of fatigue and desaturation. He reported continued home use of the RMSTD. After six weeks of outpatient therapy, he could use RMSTD at 80 cm H_2_O. During follow-up, he could perform 390 m of 6MWT with the Borg scale [7, 8] maximum of 11 while maintaining all the vitals within normal limits.

## 3. Discussion

In people living with COVID-19, the usual respiratory symptoms include dyspnea and fatigue. Many individuals with preserved lung function have reported exercise intolerance and dyspnea, which is even more common among those who are hospitalized and had ground glass opacity, lung fibrosis, and a deranged pulmonary function test. This type of individuals who are hospitalized and reported to have decreased exercise capacity (indicated by reduced 6MWT), increased respiratory drive, and exercise-induced deoxygenation may be attributed to inspiratory muscle dysfunction [1]. During the initial assessment, our patient had exertional dyspnea, breathlessness, and increased respiratory drive. Due to prolonged immobilization and continued positive airway pressure, it is quite possible that his respiratory muscles might have weakened over time due to underutilization.

**Figure 5:**
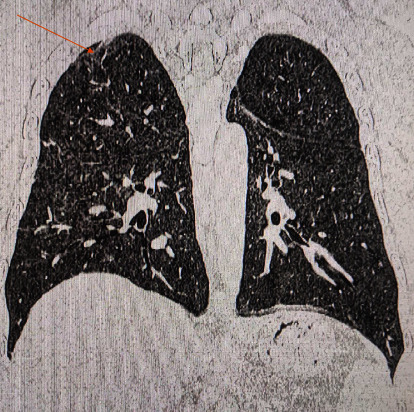
HRCT of the patient chest after completion of therapy (arrow indicates the area of fibrosis).

According to available data, individuals who were sicker at the time of their initial COVID-19 hospitalization, particularly those who required a high-flow nasal cannula and invasive or noninvasive mechanical ventilation, are more likely to develop long-term pulmonary sequelae. These sequelae include anomalies on imaging that point to pulmonary fibrosis (PF) and reduced pulmonary diffusion capabilities. All these come under post-acute COVID-19 syndrome (PACS) which is a new and significant health issue and requires enough attention [9]. These respiratory symptoms along with abnormalities in lung imaging may regress over time. However, complete resolution is not seen in many cases, as was in our case [10]. Our patient, at the end of the therapy, had a Modified Barthel Index (MBI) of 100, but still some areas of fibrosis were present (Figure 5).

Inspiratory muscle training (IMT) is a nonpharmacological intervention that has been effective in managing breathlessness in many clinical conditions. IMT can be carried out independently at home and has been shown to produce clinically meaningful improvements in dyspnea and quality of life in patients with COPD and bronchiectasis. There are several devices on the market, like spirometers, that can aid in improving sputum expectoration, preventing lung infections postsurgery, and increasing inhaled lung volume. However, the effectiveness of incentive spirometry for long-term conditions remains debated. In contrast, inspiratory muscle training plays a crucial role in reducing or preventing postoperative pulmonary complications, making it a more reliable and essential approach for long-term respiratory health [11]. Comparatively, respiratory muscle training with a trainer device appears to be more beneficial for quality of life, exercise capacity, and respiratory function in patients with pulmonary dysfunction brought on by any respiratory disease, internal neural system disorders, or disorders of the central nervous system [12–14]. Even in severe COVID-19 patients who were weaned from mechanical ventilation, the therapeutic importance of 2-week Inspiratory Muscle Training (IMT) is high. In a study which evaluated a number of clinical parameters in COVID-19 patients after they were successfully weaned from mechanical ventilation, it was shown that the patients receiving IMT sessions felt very strongly motivated to perform IMT sessions on a regular basis since they were feeling better from session to session [15]. Given that respiratory muscle weakness predicts poor outcomes following COVID-19 infection, IMT could represent a feasible initial step towards a whole-body rehabilitation program for patients with long COVID [16]. In one of the studies, IMT was found to be beneficial in managing symptoms and was associated with perceived improvements in respiratory symptoms, exercise and work capacity, and daily functioning along with beneficial action in respiratory muscle, and the study highlights the potential of IMT as part of a holistic recovery program, emphasizing the need for individually tailored rehabilitation approaches due to the complex and varied symptoms of post-COVID-19 [17]. Home-based IMT is also a safe, feasible, and effective approach for enhancing functional capacity in individuals with long COVID [18]. Trained individuals can be provided with the device for home exercise therapy, as demonstrated in this case report.

**Figure 6:**
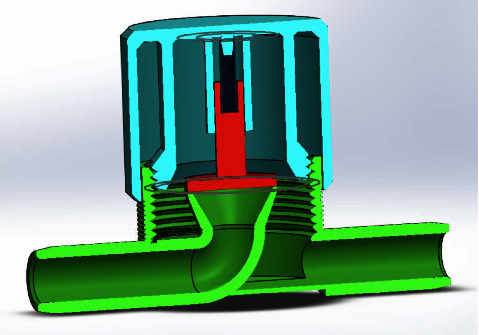
Cross-section of the 1st generation design version of the RMT device. Valve plunger in red, valve body in green, spring in dark blue, and valve cap for adjustment in light blue.

**Figure 7:**
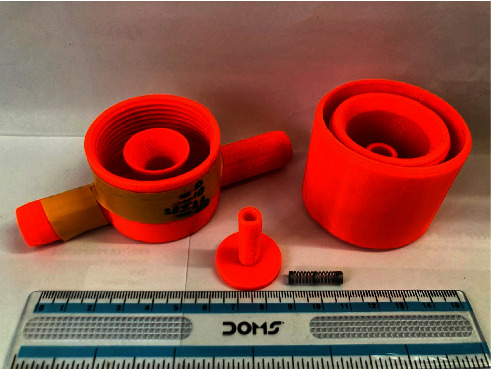
1st generation design version of the RMT device showing valve plunger, valve body, spring, and valve cap for adjustment.

**Figure 8:**
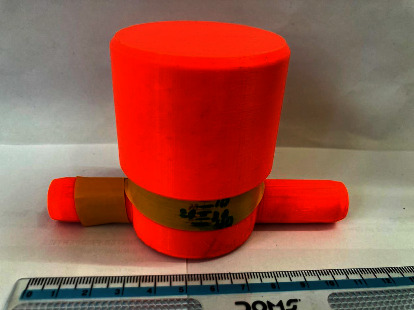
The 1st generation RMT prototype, calibrated 0 to 40 cm H2O. The yellow tape on the left inlet port indicates the port for expiratory use. Rotation of the cap results in a different maximum expiratory pressure.

As already stated, with a meticulous evaluation of the patient's condition, we opted for a comprehensive management approach that would not only provide clinical benefits but also promote psychological and economic well-being. The medical team paid attention to various clinical parameters, while the counselors built a good rapport with the patient and provided customized counseling. Collaborating with the speech and language department, we devised a pulmonary therapy plan that would enhance the overall pulmonary function of the patient, with a particular emphasis on improving their breathing pattern and strengthening their respiratory muscles. Several respiratory muscle training devices are available in the market. However, most of them are very expensive (ranging from 30$ to 100$), making it inaccessible for many patients from low- to middle-income countries, including Nepal [19]. We thus decided to develop a low-cost respiratory muscle trainer, which could be used in a low-income setting like ours. Thanks to the expertise of the biomedical engineering department of our hospital, we were able to design and produce an affordable device that is highly effective in meeting the needs of our patients. Initially, the first prototype-1^st^ Generation (Figures 6, 7, 8) of the valve stem, orifice, and spring took a significant amount of time (9 hours and 42 minutes) and material (80 grams) to print, costing around United States Dollar (USD) 2.75 (equivalent to Nepalese Rupee (NPR) 328). Further refinements resulted in a reduction in printing time to 4 hours and 5 minutes, material usage to 37.3 grams, and the final cost to USD 1.30 (NPR 157). This revised design-2^nd^ Generation (Figures 1, 2, 3) was put through the same functional tests and calibration, and it exhibited similar performance for expiratory use as the first prototype.

A large number of individuals underwent mechanical ventilation during the pandemic, and it seems imperative for tertiary care hospitals and rehabilitation centers to foresee and deal with postintensive care syndrome. A collaborative team with effort directed towards early swallowing assistance, speech therapy, and use of ventilator-compatible speaking valves can lessen the possible detrimental effects of extended invasive ventilation and postintensive care syndrome brought on by COVID-19. The ability to communicate with loved ones, eat what they want, and breathe naturally are all vital to patients [20]. Prosocial behaviors are key in promoting resilience, well-being, and quality of life during times of loss and separation [21]. Reinforcing social bonds through interventions can effectively support grieving individuals. Cultivating social connections in and after the COVID-19 pandemic is crucial for positive attitudes, enhanced well-being, and improved quality of life. Strategic recovery planning should prioritize fostering new social bonds that bring positive experiences and contribute to overall health and well-being. Adaptive interventions, such as psycho-socio, digital, and nature-based approaches, empower individuals psychologically, socially, and emotionally, improving mental, physical, emotional, and spiritual well-being [22]. These interventions have the potential to enhance well-being during and beyond the pandemic. A multidisciplinary therapeutic strategy can be employed to satisfy these needs. Early medical attention and speech therapy assistance for respiratory issues can immediately relieve the symptoms. In this regard, the use of IMT device can be very beneficial. Further research is necessary to evaluate the effectiveness of respiratory muscle training (RMT) on respiratory symptoms in individuals with long COVID. Studies examining the impact of RMT on functional outcomes such as dyspnea, cough efficacy, respiratory complications, hospital admissions, and quality of life, as well as its potential carryover effects on respiratory function, morbidity, and mortality, are crucial in establishing the benefits of this intervention. Additionally, short- and long-term studies are needed to determine optimal dosage and dose-response relationships, particularly in relation to increasing vital capacity, inspiratory volumes, and strength, which appear to be particularly impacted by RMT. The design is aiming to provide a low production volume solution for hospitals where these devices are not available or affordable and where a 3D printer and a biomedical department are at hand. As a limitation, the design and production method are not suitable for scaling up to larger volumes and commercial production, where different production technologies would be preferred. Similarly, the simplicity of the design limits both maintenance requirements and failure mechanisms, and only a simple functional test and initial calibration after manufacturing are required. Since the design aims for one device to serve a single patient, only the risk cross-contamination between patients is nihil. In the rare case, failures are detected during manufacturing or after handing out to a patient, the failed parts can simply be reproduced to correct the device.

## 4. Conclusion

COVID-19 has long-term, multisystemic sequelae, collectively grouped under post-COVID-19 syndrome and long COVID. These require proper management and rehabilitation and for individuals recovering from severe COVID-19, especially those who were weaned from mechanical ventilation, inspiratory muscle training can be beneficial for pulmonary rehabilitation. The device has a calibrated pressure valve. The pressure valve creates an isometric load on the muscles involved in coughing, swallowing, and breathing out. This is similar to the concept of lifting weights to strengthen muscles in other parts of the body. The system applies the principle used in weight machines at the gym. By providing this isometric load, the device helps strengthen the targeted muscles effectively. Through this report, we have successfully described the designing and clinical applicability of a low-cost, indigenously produced respiratory training device for pulmonary rehabilitation of long COVID. The article has certain limitations as it describes only a single case, which restricts the generalizability of the findings. To strengthen the evidence supporting this low-cost device, it would be beneficial to include multiple case reports, thereby providing a broader understanding of its effectiveness across different patients. Future research should focus on conducting case series using this device, which would allow for a more comprehensive assessment of its performance. Additionally, there is a promising opportunity to conduct a pilot study to evaluate the device's effectiveness in a larger population, further validating its potential as an accessible solution for respiratory muscle training.

## Data Availability

The clinical data that support the findings of this study are available from the corresponding author upon reasonable request.
